# Effect of Verbal Encouragement on Performance and Affective Responses in Male Sport Science Students during Sprint Modalities

**DOI:** 10.3390/sports12040108

**Published:** 2024-04-15

**Authors:** Faten Sahli, Nidhal Jebabli, Okba Selmi, Manar Boujabli, Hajer Sahli, Makram Zghibi, Monoem Haddad

**Affiliations:** 1 Research Unit: Sport Sciences, Health and Movement, UR22JS01, High Institute of Sports and Physical Education of Kef, University of Jendouba, Kef 7100, Tunisia; sehli.feten@gmail.com (F.S.); jnidhal@gmail.com (N.J.); okbaselmii@yahoo.fr (O.S.); manarboujabli2019@gmail.com (M.B.); sahlihajer2005@yahoo.fr (H.S.); makwiss@yahoo.fr (M.Z.); 2 Physical Education Department, College of Education, Qatar University, Doha P.O. Box 2713, Qatar

**Keywords:** teacher verbal encouragement, student verbal encouragement, sprint forms performance, agility, rating of perceived exertion, feeling scale

## Abstract

This investigation aimed to examine the effect of verbal encouragement teacher to student (VETS) versus verbal encouragement student to student (VESS) on physical performance and affective responses during different modalities of sprint tests in active male students. In a randomized crossover design, twenty-two male sport science students (age: 21 ± 1.2 years, body height: 1.77 ± 0.3 m, body mass: 76.6 ± 2.1 kg, BMI: 22.9 ± 1.3 kg·m^−2^) performed linear and change-of-direction sprint tests under one of three conditions: (1) VETS; (2) VETS; (3) no verbal encouragement. In each condition, participants performed the 20 m sprint test, the 10 × 2 sprint test, and the L sprint test. The assessed parameters comprised physical performance, rating of perceived exertion (RPE), and a feeling scale. Post hoc test analysis indicates a significant increase in physical performance during VETS and VESS conditions compared to the control condition due to a decrease in sprint in line 20 m (VETS: *p* < 0.001, d = 0.55; VESS: *p* = 0.016, d = 0.41), sprint 10*2 (VETS: *p* < 0.001, d = 0.64; VESS: *p* = 0.05, d = 0.36), and sprint L (VETS: *p* = 0.001, d = 1.19) times compared to the control condition. Moreover, the feeling score was greater after VETS compared to other conditions (*p* = [<0.001–0.001], d = [0.77–1.18]). In addition, the RPE had no effect on sprint performance between the different conditions. It is indicated that VETS, rather than VESS, is a more significant and effective way to increase effort intensity and positive feelings during sprinting modalities.

## 1. Introduction

Research carried out over the last decades on the effect of verbal encouragement (VE) from teachers/trainers during training sessions or even physical education and sports classes has shown the beneficial effects on physical performance for players/students [1,2]. Also, VE is commonly used by coaches, players, and spectators in different sports with a dynamic and competitive environment [3].

The ergogenic effects of VE on physical performance and psychophysiological responses have been extensively studied in several studies [4]. Particularly, a range of activities including endurance [5,6,7], change-of-direction [8], strength [9], and specific sport exercises [1] have been used to assess the potential effects of VE.

In fact, the startle mechanism, known as the defense reflex in the brainstem in response to loud sound stimuli [7], has been hypothesized to be one of the underlying reasons for improved physical performance with VE [10]. Other explanations have reported the advantages of VE for athletic performance could be attributed to the delayed afferent signals associated with neurological fatigue, which improved force production [11,12], increased arousal [10], and promoted positive mood [5]. VE, therefore, presents an interesting tool for motivating students and involving them more in exercise and in the motor skills [1,2,13,14].

In this context, previous research [12,15] demonstrated that VE was insufficient to promote improvement in physical performance, including strength and speed.

In physical education sessions, VE from a teacher is recognized as an external motivational tool that fosters positive effects on physical involvement and motivation to participate in training [1]. A physical education teacher’s verbal encouragement (VETS) is considered an external motivator that positively impacts physical commitment, engagement, and exercise desire [3]. Research indicates that the physical and affective demands of physical activity, particularly briefs efforts, are increased when physical education teachers provide motivation [16]. Furthermore, favorable emotional reactions and increased intensity may be associated with the motivation derived from training activities [17]. Numerous studies on exercise training have emphasized the significance of VETS in influencing effort intensity, as reflected in the rating of perceived exertion (RPE) and physiological responses such as heart rate (HR) and lactate concentration [La]. For example, Selmi et al. [3] found that during agility exercise with VE the values of HR, RPE, and [La] were considerably greater than those during agility exercise without VE. Bartlett et al. [18] stated that VE induces into favorable motivation (i.e., positive mood) thereby improving physiological demands during high-intensity interval running. Moreover, Aydi et al. [2] highlighted that encouragement cues from instructors lead to enhanced physical performance and a more positive emotional state during circuit training sessions in adolescent students. Moreover, Sahli et al. [19] reported that VE and compliments from the teacher to student during repeated sprint tests with change-of-direction had a favorable impact on physical performance.

Enhancing sports performance requires improvements on both physical and psychological fronts [3]. Of particular importance among students are speed exercises, which demand substantial psychomotor capabilities [20]. For example, speed and change-of-direction speed are very interesting physical qualities [21].

Theoretically, change-of-direction (COD) speed is characterized by agility movements in which a pre-planned change of direction (COD) is performed without the need to respond to a stimulus [22,23]. Various factors can influence the speed of COD. In fact, previous research indicates that lower extremity neuromuscular qualities and line sprint speed may be significant predictors of COD performance [23,24]. In a COD, rapid application of force is necessary to accelerate, and eccentric and concentric force development is necessary to decelerate and then accelerate again in the opposite direction. It is, therefore, logical to assume that, in straight line sprinting, COD performance and strength/power may be related. Few studies have evaluated the effect of VE on the performance of sprinting in line or sprinting with COD. Indeed, the study by Sahli et al. [19] reported that VE during the repeated direction change test is beneficial for improving the fast time index.

Also, recent studies have reported that students’ motivation in physical education session has a positive effect on improving the performance of brief and intense exercises [25,26]. It has been suggested lately that, in order to promote psychophysiological progress, short-term, high-intensity activities should be promoted [3].

More often, participants preferred to say that the VE was only useful, or even more so, later in the test, when they felt tired and thought about stopping [7]. Overall, the results indicate that, although regular VE is crucial, VE should improve the management of psychological and physical stress during intense exercise.

To our knowledge, no study has evaluated the effect of verbal encouragement, whether from the coach/teacher to the athlete/student or from the athletes/students to the athletes/students, on performance during the sprint in line or sprint with COD tests. Likewise, no study has presented the impact of VE with an increase in the number of changes of direction.

Therefore, the aim of this study was to examine the effect of VETS vs. VESS on physical performance and affective responses during different modalities of sprint tests in active male students. Based on the previous results [19], we hypothesized that both VE conditions could improve sprint and COD ability performance.

## 2. Materials and Methods

### 2.1. Participants

The sample size for our study was determined using the G*Power program (version 3.1.9.3, Düsseldorf, Germany). A priori power analysis, conducted using the F-test family, indicated that a sample size of 16 participants was required to detect large effects with a power of 0.8 and an alpha level of 0.5. To accommodate potential dropouts, we recruited additional students, resulting in a total sample size of 22 participants.

Twenty-two male sport science students volunteered to take part in the study (Table 1). These participants were selected because they were active in sport (6 h·wk^−1^) and had previous competitive experience in sprint racing (4 ± 2 years). The students were familiarized with the experimental protocol and were informed about the procedures. Participants were weighed without shoes and wearing as little clothing as possible, to the nearest 0.01 kg, on an electronic scale (Seca, Hamburg, Germany). A stadiometer was used to measure body height, with an accuracy of 0.1 cm (Seca, Hamburg, Germany). Participants gave written informed permission after being fully informed of the study’s objectives and possible risks. A local research ethics committee (051-2023) was carried out in accordance with the most recent Declaration of Helsinki for human evaluation.

### 2.2. Procedures

A randomized repeated measures crossover design was employed. The present study involved three experimental conditions to examine the effects of verbal encouragement (VETS, VESS, and control) on physical performance in terms of speed, change-of-direction performance, RPE, and feeling scale responses.

The research was conducted during the 2022–2023 college mid-season. Measurements of height and body weight were taken prior to the start of the experimental investigation. All measurements were taken on the same sport pitch at the same time of the day (between 8:00 a.m. and 10:00 a.m.) in order to limit the potential effects of circadian change. The week before the experimental period (familiarization sessions), all test procedures, instruments, and equipment were explained and practiced.

In the experimental period, three sessions of speed testing were performed. In each session, the participants performed physical tests (the 20 m sprint test, the 10 × 2 sprint test, and the L sprint test). There was a seven-day gap between the sessions. In random order, each condition was performed once. The RPE and FS scores were assessed following each sprint modality. All students abstained from strenuous exercise for a minimum of 48 h before testing and measurements were taken. Prior to each test intervention, participants completed a standardized 15 min warm-up, which included jogging, proprioception exercises, coordination movements, and dynamic stretching. The warm-up concluded with four 8 m sprints, with three minutes of passive recovery between the warm-up and the speed tests. Participants were acquainted with the feeling scale, RPE scale, and speed protocols before the commencement of the study.

### 2.3. Verbal Encouragement Characteristics

During the sprint tests, a physical education teacher or student was positioned on the finish line to give the auditory signal of departures with the whistle and encourage the participants using terminology common in soccer (i.e., “way to go!”, “come on!”, “good job!”, “excellent!”, “come on, push it!”, “again!”, “faster!”). Encouragement was continuous throughout the sprint and spontaneous. The instructor/student did not provide any VE during the control condition. All VE was given at a volume above normal conversational volume. All conditions were carried out in a climate without noise or sound other than the verbal encouragement.

### 2.4. Measures

#### 2.4.1. Sprint in Line 20 m

The sprint test comprised a 20 m track with split time recording every 20 m. Every student completed two trials, with a minimum of three minutes of rest in between. The sprint score was determined by taking the best result from the three trials. Two sets of photocells were positioned 20 cm from the starting line and 20 cm from the finish line. Electronic photocell gates were used to assess test performance (Globus, Microgate, Bolzano, Italy) with an accuracy of 0.01 s. The RPE and FS were measured just after the repeated sprint test.

Pilot data from 22 participants collected on two different days (familiarization sessions) were used to determine the reproducibility of the test (intraclass correlation coefficient [ICC] = 0.997).

#### 2.4.2. Sprint 10 × 2

Each student initiated a linear sprint from the starting line, covering a distance of 20 m. Upon reaching the designated point, they touched a line on the floor with one foot and swiftly executed a 180-degree change of direction (COD) before returning to the starting line as rapidly as possible. Test performance was assessed using electronic photocell gates (Globus, Microgate, Italy) positioned at the start–finish points, approximately 0.5 m above the ground, with a precision of 0.01 s. The RPE and FS were evaluated immediately following the repeated sprint test [27]. Pilot data from 22 participants collected on two different days (familiarization sessions) were used to determine the reproducibility of the test (ICC = 0.990).

#### 2.4.3. Sprint L

**Figure 1:**
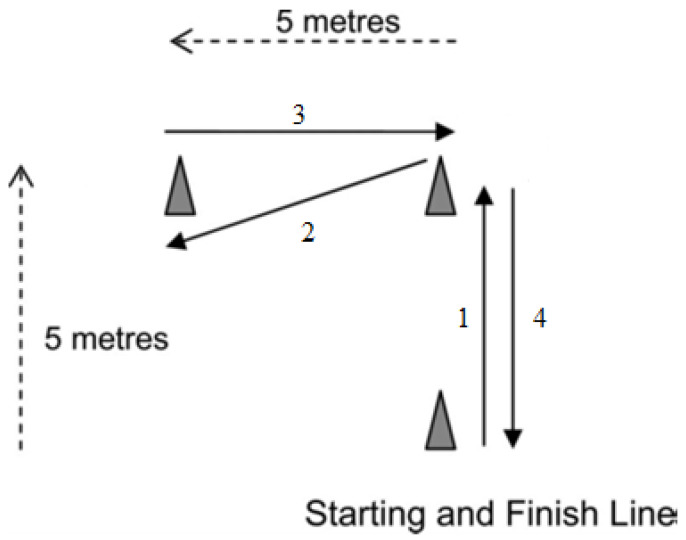
Schematic illustration of the L run test. 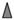 cones. 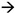 sprint direction.

Three cones were positioned 5 m apart for the L-shaped track (Figure 1) [28]. The total distance traveled was equal to 20 m. Each student performed two trials with a minimum of three minutes recovery between the tests. The best performance obtained after three trials was used as the sprint score of the L test. One pair of the electronic timing system sensors was set 20 cm above the floor, 3 m apart, and facing each other at the start–finish line. Electronic photocell gates were used to assess test performance (Globus, Microgate, Italy) with an accuracy of 0.01 s. The RPE and feeling scale (FS) were measured just after the repeated sprint test. Pilot data from 22 participants collected on two different days (familiarization sessions) were used to determine the reproducibility of the test (ICC = 0.988).

#### 2.4.4. Rating of Perceived Exertion

The Ratings of Perceived Exertion (RPE) scale [29], ranging from 6 to 20, was administered immediately after each sprint, including the 20 m sprint test, the 10 × 2 sprint test, and the L sprint test. This scale aimed to assess the students’ intrinsic intensity levels. Participants were asked, “How was your workout?”, in order to determine their RPE. The RPE has been widely verified and utilized in various scientific studies [30], thus establishing its validity and reliability.

#### 2.4.5. Feeling Scale

The feeling scale [31] was employed to assess affective valence, representing feelings of pleasure or displeasure. This 11-point bipolar scale features verbal anchors ranging from “very good” (+5) to “very awful” (−5). Students were instructed to exercise until they reached a “good” feeling. Previous research has validated the FS as a reliable measure of affect [31].

### 2.5. Statistical Analysis

Data analyses were conducted using the Statistical Package for the Social Sciences (SPSS) version 26.0 (SPSS Inc., Chicago, IL, USA). Descriptive statistics, including means and standard deviations (SDs), were utilized to present the data. The Shapiro-Wilk test was employed to assess the normality of the data distribution. The reliability of test–retest for all variables was evaluated using the ICC model proposed by Cronbach. One-way analysis of variance (ANOVA) was performed to detect differences between conditions for the 20 m sprint, 10 × 2 sprint test, and L test. Bonferroni-adjusted post hoc testing was conducted following significant differences. Cohen’s d (d) was calculated to determine the magnitude of meaningful differences in the data, with classifications of trivial (<0.2), small (0.2–0.59), medium (0.60–1.19), large (1.2–1.99), and very large (≥2.0) [32]. Statistical significance was predetermined at *p* < 0.05.

## 3. Results

### 3.1. Fitness Performance

**Figure 2:**
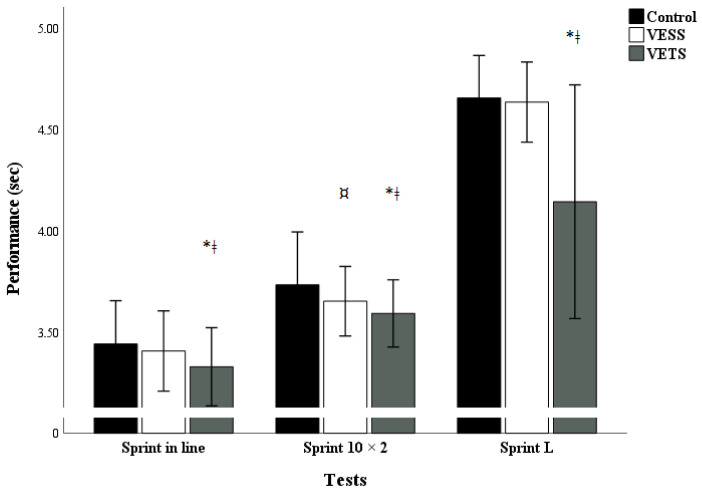
Effect of different verbal encouragement conditions on physical performance. Note: Mean ± SD; VESS: verbal encouragement student to student; VETS: verbal encouragement teacher to student; * significant difference between VETS and control (*p* < 0.05); ǂ significant difference between VETS and VESS (*p* < 0.05); ¤ significant difference between VESS and control (*p* < 0.05).

The test results of VE type on speed and change-of-direction performance are outlined in Figure 2.

Post hoc test analysis indicates a significant decrease in sprint in line 20 m, sprint 10*2, and sprint L times during the VETS condition (sprint 20 m: *p* < 0.001, d = 0.55; sprint 10*2: *p* < 0.001, d = 0.64, sprint L: *p* = 0.001, d = 1.19), more than VESS (sprint 10 × 2: *p* = 0.05, d = 0.36) compared to the control condition. However, no significant change was observed between the VESS and VETS for 10 × 2 tests.

### 3.2. Feeling Scale and Perceived Exertion

**Figure 3:**
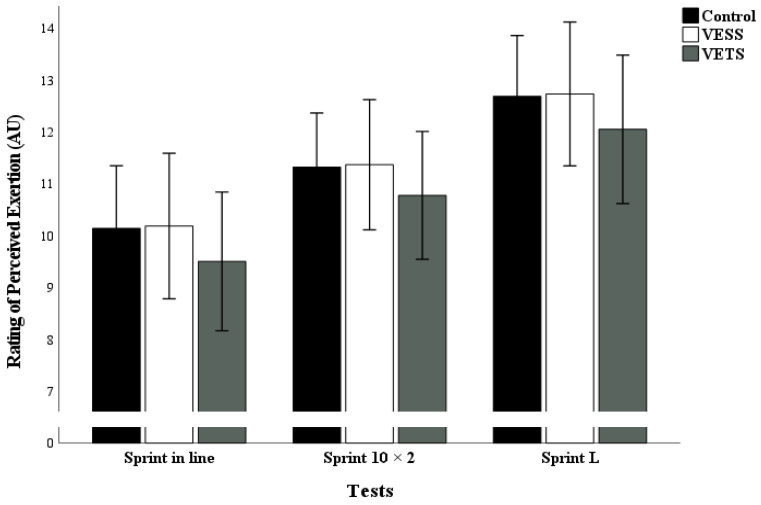
Effect of different verbal encouragement conditions on RPE post-test. Note: Mean ± SD; VESS: verbal encouragement student to student; VETS: verbal encouragement teacher to student.

**Figure 4:**
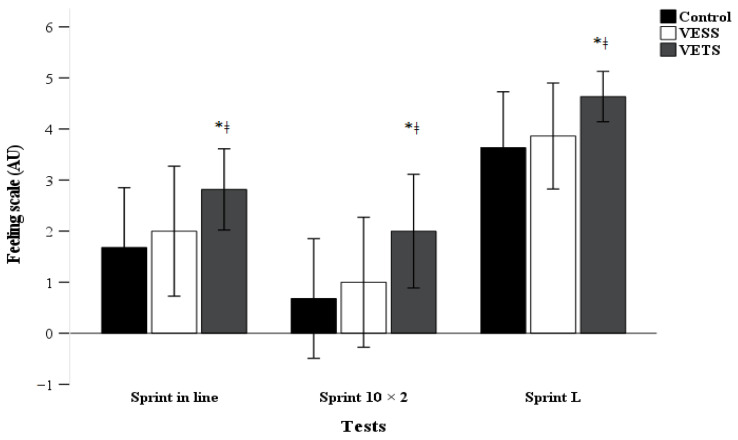
Effect of different verbal encouragement conditions on feeling scale post-test. Note: Mean ± SD; VESS: verbal encouragement student to student; VETS: verbal encouragement teacher to student. * Significant difference between VETS and control (*p* < 0.05); ǂ significant difference between VETS and VESS (*p* < 0.05).

The test results of VE type on RPE and FS are outlined in Figure 3 and Figure 4.

Post hoc test analysis indicates a significant increases in FS during all test in the VETS condition (sprint 20 m: *p* = 0.001, d = 1.14; sprint 10 × 2: *p* < 0.001, d = 1.16; sprint L: *p* < 0.001, d = 1.18), compared to control condition. However, no significant difference occurred between VESS and the control condition (*p* > 0.05). In addition, no significant difference occurred between conditions for RPE.

## 4. Discussion

This study aimed to examine the effect of VETS and VESS on physical performance on the RPE and affective aspects during exercise of active male student during sprint exercises (line 20 m, sprint 10 × 2, and sprint L). The results showed the following: (1) VETS results increased more for the sprint in line 20 m, sprint 10 × 2, and sprint L times than that of VESS and control conditions; (2) the feeling score was greater after VETS compared to other conditions; (3) the RPE has no effect on sprint performance between the different conditions.

Regarding physical responses, this study indicates that sprint performance has been significantly improved during VETS. This implies that the pupils performed the exercises with great effort, which led to a high level of physical demand [3]. The VE variable decreases the time of sprints. These findings suggest that VETS can motivate students to put forth their best effort and show a strong commitment to participating in physical activity. This finding is consistent with prior research demonstrating that athletes’ physical performance is enhanced by a sports teacher’s or coach’s VE [1,2,3,14,19]. For example, Edwards et al. [33] indicated that VE inspired by participants, could improve their anaerobic exercise performance. According to Selmi et al. [34], VE is crucial for enhancing physical effort during repeated agility training. This result agrees with Sahli et al. [19], who examined the effect of VE on the performance of the repeated change-of-direction (RCOD) sprint test in male students in secondary school. It was demonstrated that the fatigue index, fast time, average time, and total time were lower with VE compared to without VE [19]. Similarly, Aydi et al. [2] investigated the impact of VE on physical performance during a physical–technical training circuit. Their findings revealed that the distance covered and HR values were higher during exercises with VE compared to those without VE [2]. Furthermore, during maximal activity testing, VE every 20 s extended time to exhaustion more than VE every 60 or 180 s, according to Midgley et al. [7].

Regarding internal intensity responses, this study indicates that the RPE was stable during the sprints with different conditions. The fact that VETS increased sprint performance in line or with COD without influencing RPE could argue that VETS distracted students from the higher intensity of the exercise. In other words, the lack of significance of RPE values could be explained by the fact that VETS and VESS play a role in distracting sensory and physical signals during exercise. However, the result of the present study diverges with previous studies which reported that the psych-physiological characteristics of football players can be influenced by the presence of a physical education teacher (i.e., VE) [2,16,19]. For example, Selmi et al. [3] discussed the value and efficacy of VE in raising internal intensity during repeated agility speed training (RAS). They investigated the effects of verbal encouragement (VE) on players’ internal intensity during repeated agility sprints (RAS), revealing that the internal intensity score was higher during exercises with VE compared to those without VE. Additionally, Aydi et al. [2] examined the effect of VETS on RPE and physiological responses during a circuit for physical-technical training in adolescent students. Their findings indicated that RPE and HR values were higher during exercises with VE compared to those without VE. Furthermore, Rampinini et al. [35] explored the impact of verbal encouragement provided by coaches on perceived exertion and physiological aspects in small-sided games. The results demonstrated that HR and [La] levels were higher during small-sided games with VE compared to those without VE.

Research on affective valence is increasingly using the FS, which measures the positive affective response induced by participating in a certain activity [16,35,36,37]. In this investigation, the perceived positive affective score, which was assessed following the VETS condition, was higher than the scores that decelerated following the VESS and control conditions. Our findings suggest that VETS had a positive influence on students’ pleasant affective response during sprint activities, which is consistent with previous studies that revealed that participants who synchronized with VE experienced higher levels of satisfaction [19,38]. Sahli et al. [19] indicated that increased pleasure during exercise training may be associated with increased energy and improved cognitive performance. The results of the present study suggest that the motivation provided by the physical education teacher improved the affective aspects of the students. Indeed, Faro et al. [39] emphasized that, in order to enhance the teaching–learning process, it is critical to take students’ motivation into account as they learn. When it comes to sports performance and academic learning, motivation becomes crucial. Research in this area indicates that pupils’ levels of emotion have an impact on their motivation [36,39].

It has been proposed that the absence of change in affective valence, internal intensity, and sprint performance during exercises with VESS and under control circumstances correlates with low motivation and low enjoyment, which are connected to negative emotional perceptions. These results suggest that teacher-provided motivation during exercises can lead to improvements in physical performance and positive mood state compared to unmotivated exercises.

Overall, according to the results of the present study, physical education teachers have a greater moral and psychological influence than that of partners, which explains the important role of teacher encouragement on the physical performance and perceptual responses of students.

Several limitations of the current study warrant consideration. Firstly, the number of students was constrained due to the challenges associated with recruiting a large cohort of homogeneous participants. Additionally, the sample comprised only one age cohort of adolescent students, potentially limiting the generalizability of the findings. Furthermore, it is important to note that the study was conducted within PE sessions rather than within a long-term learning program, such as a comprehensive learning cycle. Finally, further research is required to examine the effect of VE on aerobic and anaerobic performance based on age, the quality of the study population, and gender, which will provide a baseline for future research.

## 5. Conclusions

The findings of this study suggest that VE provided by teachers significantly enhances student motivation, resulting in improvements in sprint times, internal intensity, and affective valence. Specifically, student performance improved, exercise intensity increased, and FS scores were elevated during sprints with VETS. Thus, VETS is recommended as a valuable and effective technique for increasing effort levels and fostering positive emotions during sprinting modalities. These results suggest that, to enhance participants’ motivation and physical engagement during physical training, coaches and sports teachers should incorporate VE more frequently.

## Figures and Tables

**Table 1:** Mean (±SD) values of participants’ characteristics.

Participants	Age (Years)	Body Mass (kg)	Body Height (m)	BMI (kg m^−2^)
22 sport science students	21 ± 1.2	76.6 ± 2.1	1.77 ± 0.3	22.9 ± 1.3

BMI: body mass index.

## Data Availability

This article contains all of the information that was examined during this work.
